# The role of ubiquitination and deubiquitination in urological tumours

**DOI:** 10.3389/fphar.2025.1532878

**Published:** 2025-07-23

**Authors:** Lifeng Gan, Peiyue Luo, Junrong Zou, Wei Li, Qi Chen, Le Cheng, Fangtao Zhang, Haidong Zhong, Liying Zheng, Biao Qian

**Affiliations:** ^1^The First Clinical College, Gannan Medical University, Ganzhou, Jiangxi, China; ^2^Department of Urology, The First Affiliated hospital of Gannan Medical University, Ganzhou, Jiangxi, China; ^3^ Key Laboratory of Urology and Andrology of Ganzhou, Ganzhou, Jiangxi, China; ^4^Department of Graduate, The First Affiliated Hospital of Gannan Medical University, Ganzhou, Jiangxi, China

**Keywords:** ubiquitination, deubiquitination, urinary system, molecular mechanisms, targeted therapy

## Abstract

The ubiquitin (Ub) system has been demonstrated to play a crucial role in various cellular processes, including immune responses, cell development, and programmed cell death. Ubiquitination, a form of post-translational modification, occurs in eukaryotic cells and involves several key components, such as Ub-activating enzymes, Ub-binding enzymes, and Ub-protein ligases. Recently, deubiquitinating enzymes—proteases that reverse the modification of proteins by removing Ub or Ub-like molecules, or by remodeling Ub chains on target proteins—have been identified as significant regulators of ubiquitination-mediated degradation. These enzymes profoundly influence cellular pathways and numerous biological processes, including the DNA damage response and DNA repair mechanisms. Recent studies increasingly demonstrate a relationship between ubiquitination, deubiquitination, and urinary diseases. The roles of these processes in urinary diseases are complex, encompassing various aspects of signaling, protein stability, and cellular metabolism. As research advances, the specific mechanisms by which these processes influence urologic diseases will be further clarified. This review examines recent discoveries in this field, aiming to provide new strategies and targets for the diagnosis and treatment of urologic diseases.

## Introduction

**FIGURE 1:**
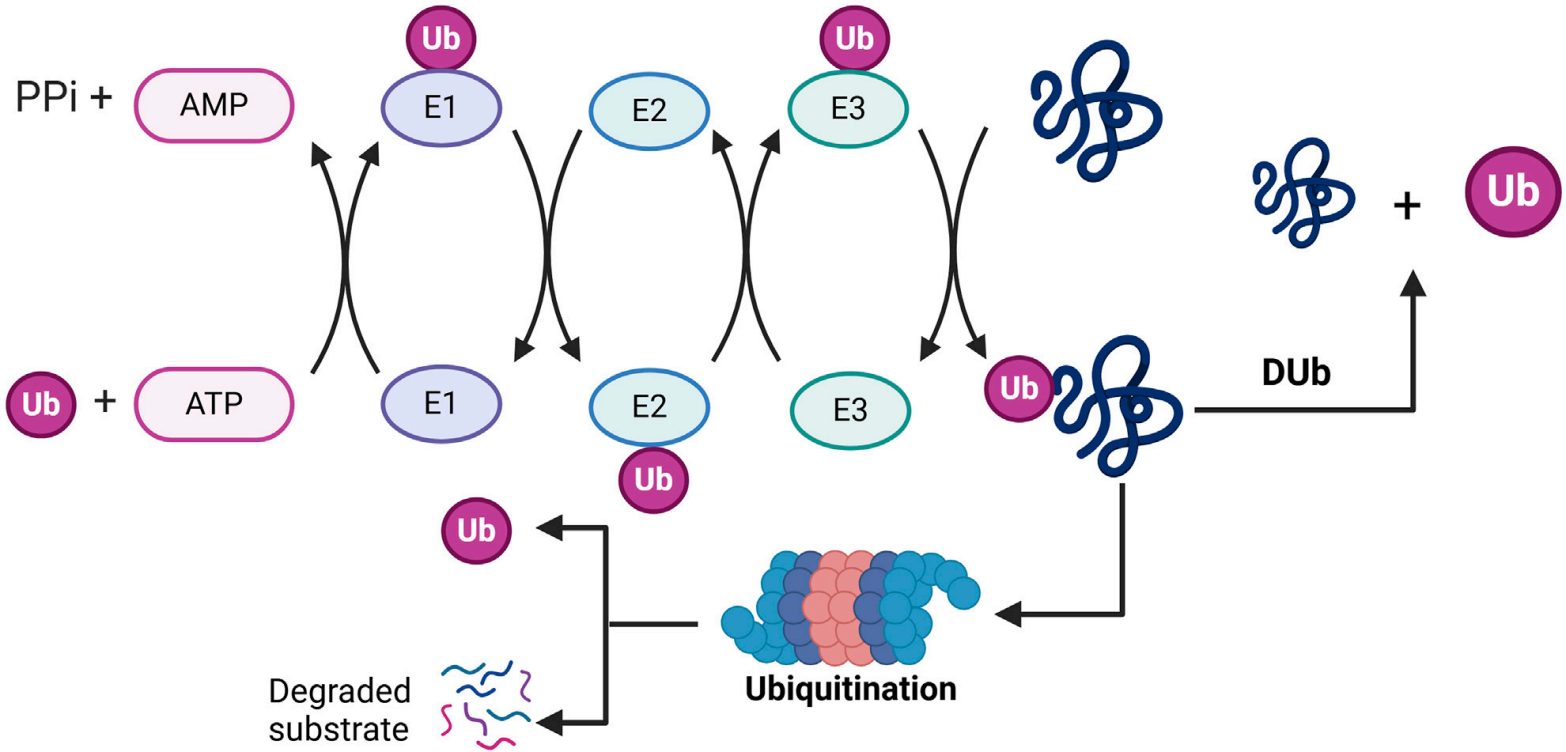
Molecular mechanisms by which ubiquitination and deubiquitination regulate substrate degradation: in the ubiquitination phase (left), ubiquitin-activating enzyme (E1) consumes ATP, attaches ubiquitin (Ub) to itself *via* a thioester bond, and subsequently transfers it to ubiquitin-conjugating enzyme (E2), where ubiquitin conjugating enzyme (E3) covalently binds the ubiquitin to substrate proteins to form a multimeric ubiquitin chain (Ub+) that labels the substrate and is degraded by the proteasome (Degraded substrate). In the deubiquitination phase (right), deubiquitinating enzymes (Dub) shear the ubiquitin chain, reversing the ubiquitination process and maintaining substrate protein stability. PPi, pyrophosphoric acid; Ub, ubiquitin; Dub, deubiquitination.

Proteins are synthesized from the genetic code encoded in DNA and are subsequently translated into polypeptide chains. These proteins can undergo modifications through chemical alterations known as post-translational modifications (PTMs) (Chen and Tsai, 2022; Singh and Ostwal, 2019). PTMs have been shown to regulate protein function by influencing various aspects of protein behavior, including activity, localization, stability, and interactions with other proteins (Mowen and David, 2014). Common PTMs include methylation, acetylation, ubiquitination, sumoylation, glycosylation, and phosphorylation, each involving the attachment of specific chemical groups or protein modifiers. These modifications impact localization, stability, interactions, folding, and gene expression (Bhat et al., 2021; Narita et al., 2019; Rape, 2018; Yang and Qian, 2017). PTMs play a critical role in regulating essential biological processes, such as cellular signal transduction, cell signaling pathways, cellular metabolism, differentiation, metastasis, the cell cycle, and proliferation (Chen T. et al., 2024). Ubiquitination, a specific type of PTM, involves the covalent attachment of ubiquitin to a target substrate, thereby affecting the substrate protein’s function by regulating its localization and stability (Popovic et al., 2014). Ubiquitination is an ATP-dependent cascade reaction that covalently attaches ubiquitin, a protein composed of 76 amino acids and expressed throughout various tissues, to a substrate protein (Hershko and Ciechanover, 1998). Initially, ubiquitin-activating enzymes (E1s) bind to ubiquitin for activation and subsequently transfer the activated ubiquitin to ubiquitin-conjugating enzymes (E2s). Finally, ubiquitin ligases (E3s) facilitate the transfer of ubiquitin from E2 to the substrate (Hershko and Ciechanover, 1998). Ubiquitination modifications of substrate proteins are recognised and degraded by the proteasome machinery (Chen X. et al., 2024; Berndsen and Wolberger, 2014), with E3 ligases playing a crucial role in the ubiquitination process due to their substrate specificity. The human genome contains approximately 1,000 E3 ligases, which can be categorized into three main groups: those homologous to E3s containing the E6AP C-terminal (HECT) structural domain, the RBR family of E3s, and the really interesting E3s that possess the RING finger structural domain (Tang et al., 2019). Deubiquitinating enzymes (DUB) remove ubiquitin molecules and reverse the process of ubiquitination to precisely regulate protein stability or function (Antao et al., 2020) (Figure 1). Deubiquitinating enzymes (DUBs) encompass six primary classes: ubiquitin-specific proteases (USPs), which modify ubiquitin chains through cysteine activity; ovarian tumour proteases (OTUs), which selectively cleave specific chain types (e.g., K48); ubiquitin C-terminal hydrolases (UCHs), which remove ubiquitin precursors; the Machado-Josephine Disease Structural Domains (MJD) family, which targets long-chain modifications; JAMM/MPN + metalloproteinases (zinc-dependent, e.g., PSMD14) that specifically process K63 chains; and the MINDY family, which preferentially degrades K48 long chains. These families work synergistically to regulate the reversibility of ubiquitin signaling, influencing critical processes such as protein degradation and DNA repair (Harrigan et al., 2018; Mevissen and Komander, 2017). Among these deubiquitinating enzymes, USPs represent the largest family, responsible for cleaving ubiquitin from its substrates. Dysregulation of USPs is linked to a range of diseases, including neurodegeneration, inflammation, and cancer (Parihar and Bhatt, 2021; Do and Baek, 2021; Liu J. et al., 2021). It is evident that maintaining a balance between ubiquitination and deubiquitination is crucial for sustaining appropriate protein levels and their functions (Snyder and Silva, 2021).

Prostate, kidney, and bladder cancers (BCa) represent the most prevalent tumors within the urinary system (Hashemi et al., 2022). Urologic tumors account for approximately 10% of the total incidence of tumors, with prostate cancer constituting 29% of cancer cases among men (Siegel et al., 2023). According to the American Cancer Society, it is projected that there will be up to 169,360 new cases of urologic tumors in the United States in 2024, resulting in an estimated 32,350 deaths (Siegel et al., 2024). These tumors are a significant cause of mortality among men globally (Safiri et al., 2021). Currently, the primary treatments for urologic tumors include surgery, radiation therapy, and targeted therapy; however, these cancers are often inadequately monitored and treated (Teo et al., 2019). A growing body of evidence suggests that a deeper understanding of the underlying molecular mechanisms of urologic malignancies may provide a promising approach to enhance therapeutic strategies and improve prognostic outcomes (Chen and Lu, 2015; Rosellini et al., 2023). In this context, the emerging roles of ubiquitination and deubiquitination in the regulation of oncogenes or tumour suppressors provides a novel and promising avenue for research. Ubiquitination regulates the activation and inhibition of tumor-associated signaling pathways (e.g., Hippo, HIF-1α) by tagging substrate proteins with E3 ubiquitin ligases, leading to their degradation or alteration of activity *via* the proteasome (Wang et al., 2022). Conversely, deubiquitination is mediated by deubiquitinating enzymes (DUBs), which stabilize pro-carcinogenic proteins (e.g., AURKA, PKM2) through the removal of their ubiquitin chains, thereby enhancing the proliferation, migration, and invasion capabilities of tumor cells (Wang et al., 2023). Additionally, both processes influence tumor immune escape by regulating the stability of immune checkpoint proteins (e.g., PD-L1) and provide energy to support tumor growth through metabolic reprogramming (e.g., activation of glycolysis) (Hu et al., 2021; Sun et al., 2020). Recent studies have indicated that ubiquitination and deubiquitination are implicated in various pathologies, including prostate (Liang et al., 2018), bladder (Shen et al., 2024), and kidney cancers (Dushukyan et al., 2017). Furthermore, our findings indicate that these processes also play a crucial role in the development and progression of other diseases affecting the urinary system, such as chronic kidney disease (Zeng et al., 2024), kidney injury (Shen et al., 2021), and renal fibrosis (Saritas et al., 2019). In this review, we examine the role of ubiquitination in the development and progression of urological diseases. Additionally, we discuss the role of deubiquitination in the regulation of these conditions. We also describe compounds that target ubiquitination and deubiquitination to influence the progression of urinary diseases. Finally, we outline the challenges and future prospects for targeting these processes in the treatment of patients with urological diseases.

## The role of ubiquitination in prostate cancer

### SPOP E3 ubiquitin ligase in prostate cancer

Cullin-Ring ligases (CRLs) represent the largest family of ubiquitin E3 ligases and play a crucial role in regulating numerous essential cellular processes, including cell cycle progression (Petroski and Deshaies, 2005). Based on the Cullin scaffolding proteins—Cullin1, 2, 3, 4A, 4B, 5, and 7—CRLs are categorized into seven subfamilies, designated as CRL1 through CRL7 (Petroski and Deshaies, 2005; Wang et al., 2014). Recently, the CRL3 subfamily complex has emerged as a significant regulator of various cellular processes, with disruptions in this degradation pathway linked to a range of human diseases, including neurodegeneration and cancer (Genschik et al., 2013). Structurally, CRL3 comprises the scaffolding protein Cullin 3, the RING protein RBX1, and one of the numerous BTB structural domain interface proteins that facilitate the recruitment of protein substrates for polyubiquitination (Genschik et al., 2013). SPOP (spotted POZ protein), a substrate-interacting junction protein within the Cullin 3-based E3 ubiquitin ligase complex, exhibits a mutation rate of 10%–15% and is a critical molecular feature of prostate cancer (PCa) (Wang Z. et al., 2020; Liu D. et al., 2021). Notably, these SPOP mutations are predominantly located in the substrate-binding MATH structural domain (Cheng et al., 2018). PCa-associated missense mutations in the MATH domain of SPOP impair substrate binding and ubiquitination, resulting in the upregulation of oncogenic substrate levels and enhanced PCa cell proliferation and invasion. This suggests a potential tumor-suppressor role for SPOP in PCa. Prostate leucine zipper (PrLZ), a member of the tumor protein D52 (TPD52) family, serves as a significant prostate-specific and androgen-responsive oncogene implicated in the malignant progression of PCa (Wang et al., 2004). In their study, Fan et al. demonstrated a direct interaction between SPOP and PrLZ using a pull-down assay. SPOP interacts with the unique N-terminus of PrLZ, while ERK1/2, a member of the mitogen-activated protein kinase family, plays a vital role in signal transduction and cancer progression (Sun et al., 2015). The research by Fan et al. indicates that ERK1/2-mediated phosphorylation of PrLZ at the Ser40 site stabilizes PrLZ by disrupting its binding to SPOP, thereby facilitating the proliferation and migration of prostate cancer cells (Table 1) (Fan et al., 2022). Heterogeneous nuclear ribonucleoprotein K (HnRNPK) is a nucleic acid-binding protein that regulates a wide range of biological events. HnRNPK is a specific member of the heterogeneous nuclear ribonucleoprotein (HnRNP) family and is involved in various cellular processes occurring in both the nucleus and cytoplasm. In addition to sharing functions with other HnRNPs, HnRNPK plays a critical role in regulating DNA transcription, pre-mRNA processing, and translation, particularly during oncogene expression (Lu and Gao, 2016; Gallardo et al., 2020). These characteristics contribute to HnRNPK’s multiple roles in the cell cycle, apoptosis, and tumor metastasis (Li et al., 2018). In the study conducted by Wu et al., SPOP was found to promote HnRNPK degradation in PCa cells in a dose-dependent manner. Furthermore, SPOP influences PrCa cell proliferation by regulating HnRNPK abundance through the post-translational ubiquitin-proteasome pathway (Wu et al., 2021).

**TABLE 1:** Ubiquitination enzymes regulate the development and progression of prostate cancer.

E3s/E2s	Starting factor	Pro/Anti tumor	Ubiquitination substrate	Biochemical function	Outcomes	References
E3	SPOP	Anti-tumor	PrLZ		Inhibits cell proliferation and migration	Fan et al. (2022)
E3	SPOP	Anti-tumor	HnRNPK		Influence on PrCa cell proliferation	Wu et al. (2021)
E3	SPOP	Anti-tumor	ATF2		Promotes cell proliferation, migration and invasion	Ma et al. (2018)
E3	SPOP	Anti-tumor	Cdc20		Affects cell viability and apoptosis	Wu et al. (2017a)
E3	SPOP	Anti-tumor	c-MYC		prostatic intraepithelial neoplasia	Geng et al. (2017)
E3	SPOP	Pro-tumor	ERG		Promotes cell migration and invasion	Gan et al. (2015)
E3	TRAF6	Pro-tumor	PI3K		Prostate cancer cell migration	Hamidi et al. (2017)
E3	TRAF6	Anti-tumor	JARID1B	K63 polyubiquitin chain	Impact on migration and recurrence	Lu et al. (2015)
E3	TRAF6	Pro-tumor	EZH2	K63 polyubiquitin chain	PCa progression, distant metastasis, and CRPC drive	Lu et al. (2017)
E3	TRAF4	Pro-tumor	TrkA	K27 And K29 polyubiquitin chain	Prostate cancer cell invasion	Singh et al. (2018)
E3	TRAF4	Pro-tumor	AR	K27 polyubiquitin chain	Promote CRPC development	Singh et al. (2023)
E3	MDM2	Pro-tumor	p53		Impact on encroachment and migration	Yan et al. (2023)
E3	MDM2	Pro-tumor	AGPS		Inhibition of prostate cancer cell proliferation	Zhang et al. (2024)
E3	TRIM36	Anti-tumor	HK2	lys48 polyubiquitin chain	Activates iron death and inhibits NED	Zhao et al. (2023)
E3	TRIM32	Pro-tumor	STIM1		Promotes PCa cell transfer	Zhou et al. (2023)
E3	TRIM21	Anti-tumor	P62	K63 polyubiquitin chain	Inhibits apoptosis and promotes PCa bone metastasis	Tang et al. (2024)
E3	FBXW2	Anti-tumor	EGFR		Inhibits PCa cell proliferation and metastasis	Zhou et al. (2022)
E3	FBXO11	Anti-tumor	β-catenin		Affects EMT and metastasis	Xuan et al. (2024)
E3	FBXO45	Pro-tumor	Par-4		Affects cell cycle, apoptosis	Ganapathy et al. (2022)
E3	AHR	Anti-tumor	AR		Affects PCa cell growth and proliferation	Chen et al. (2020)
E3	Siah2	Pro-tumor	AR		Influence on prostate cancer cell proliferation	Jing et al. (2018)
E3	SKP2	Pro-tumor	FOXA1	K6 And K29 polyubiquitin chain	Contributed to PCa genesis and development	Celada et al. (2023)
E3	COP1	Pro-tumor	STAT3		Promoting prostate cancer aggression	Dallavalle et al. (2016)
E3	TBL1X	Pro-tumor	TP53		Influence on immune evasion	Gong et al. (2023)
E3	ASB1	Anti-tumor	CHCHD3	K48 polyubiquitin chain	Promoting prostate cancer cell proliferation *in vitro*	Zhao et al. (2024)
E2	UBE2N	Pro-tumor	Axin1		Inhibition of cell viability and glycolysis in prostate cancer cells	Yang et al. (2024)

Activating transcription factor 2 (ATF2) is a member of the ATF/CREB bZIP family of transcription factors that heterodimerizes with members of the JUN and FOS transcription factor families (Lopez-Bergami et al., 2010). SPOP interacts with ATF2 *in vivo via* the N-terminal substrate-bound MATH domain, and ATF2 has been identified as a *bona fide* substrate of the SPOP-CUL3-RBX1 E3 ubiquitin ligase complex. Impaired binding of SPOP mutants to the ATF2 protein leads to reduced proteasomal degradation and subsequent accumulation of ATF2 in prostate cancer cell lines and specimens. This accumulation contributes, in part, to SPOP inactivation-induced prostate cancer cell migration and invasion (Ma et al., 2018). Additionally, there is evidence that SPOP specifically interacts with proteins through its N-terminal MATH structural domain in the context of prostate cancer. Recent studies have begun to unveil the potential oncogenic activity of Cdc20 (Kidokoro et al., 2008), with genetic ablation of Cdc20 primarily resulting in elevated apoptosis (Manchado et al., 2010). High expression of Cdc20 is strongly correlated with advanced clinical stages and poor prognosis in various human cancers, including prostate cancer (Mao et al., 2016). The E3 ligase function of SPOP serves as a novel Cdc20 ligand by facilitating the polyubiquitylation of Cdc20, leading to its subsequent degradation *via* the 26S proteasome. Thus, the SPOP E3 ligase acts as a novel negative regulator of Cdc20, which in turn influences cell viability and apoptosis in prostate cancer (Wu F. et al., 2017). c-MYC proteins have been identified as novel substrates of SPOP, while Cul3/Rbx1 serves as a new E3 ligase system for c-MYC, playing a crucial role in the regulation of proliferation and c-Myc expression in prostate luminal epithelial cells. In this context, SPOP directly binds to the c-Myc protein, facilitating its ubiquitination and subsequent degradation (Geng et al., 2017). Additionally, fusions of proto-oncogenes to strong promoters or enhancers can lead to the upregulation of mRNA levels. A notable example is the E26 translationally specific (ETS) family of transcription factor fusions in prostate cancer (Kumar-Sinha et al., 2008), with the TMPRSS2-ERG fusion being the most prevalent, occurring in approximately 50% of prostate cancer cases (Tomlins et al., 2005). SPOP has been reported to specifically interact with ERG, promoting its ubiquitination and degradation through WT-SPOP, which negatively regulates ERG-mediated prostate cancer cell migration and invasion (Gan et al., 2015).

## TRAF proteins in prostate cancer: TRAF6/TRAF4

TRAF is a class of cytoplasmic junction proteins, comprising six classical members (TRAF1-TRAF6) and one non-classical member (TRAF7) in mammals. The TRAF family of proteins plays a significant role in signaling mediated by the TNFSF and TLR/ILR receptor superfamilies, regulating the activation of various signaling pathways, including mitogen-activated protein kinase (MAPK) (Kashiwada et al., 1998). Additionally, the TRAF family is implicated in critical cellular processes such as proliferation, differentiation, survival, and apoptosis, as well as in immune and inflammatory responses (Bradley and Pober, 2001). The N-terminal region of TRAF6 contains a RING finger domain and five zinc fingers; the RING domain is responsible for its E3 ubiquitin ligase activity, while the zinc fingers primarily provide structural support for the RING domain’s function (Lamothe et al., 2008). At the C-terminal end, there exists a TRAF structural domain composed of a convoluted helix and a conserved TRAF-C domain (Rothe et al., 1994). This C-terminal TRAF domain facilitates important biological functions by mediating self-binding interactions with receptors and other signaling proteins that act upstream of TRAF6 (Wajant et al., 2001). Recently, TRAF6 has been found to play a role in ubiquitination processes in prostate cancer. Specifically, transforming growth factor-β (TGF-β) activation is dependent on phosphatidylinositol 3′-kinase (PI3K), with TRAF6 polyubiquitinating p85α, the regulatory subunit of PI3K. This action promotes the formation of a complex between the TGF-β type I receptor (TβRI) and p85α, leading to the activation of PI3K and AKT, which subsequently influences the migration of prostate cancer cells (Hamidi et al., 2017). Abnormal elevations of JARID1B and histone H3 lysine four trimethylation (H3K4me3) are frequently observed in various diseases, including PCa. Their expression has been associated with TRAF6, as demonstrated in a study by Lu et al. SKP2 (S-phase kinase-associated protein-2) serves as the E3 ligase and F-box protein component of the SKP2 SCF complex (Skp1-Cul1-F-Box), which triggers the ubiquitin-mediated degradation of p27 and other proteins (Carrano et al., 1999). In the study, SKP2 modulated H3K4me3 levels by regulating TRAF6-mediated K63-linked ubiquitination of JARID1B, a key demethylase of H3K4me3, thereby influencing prostate cancer migration and recurrence (Lu et al., 2015). Additionally, Lu et al. reported that SKP2 and TRAF6 are involved in another mechanism of ubiquitination in prostate cancer (Ngollo et al., 2014). The levels of SKP2 and zeste homologue enhancer 2 (EZH2) are highly correlated with the aggressive features of human PCa. Their findings indicate a positive correlation between SKP2 and EZH2, with SKP2 stabilizing EZH2 by reducing TRAF6-mediated K63-linked ubiquitination of EZH2 (Lu et al., 2017). Importantly, EZH2 plays a critical role in PCa progression, distant metastasis, and castration-resistant prostate cancer (CRPC) (Min et al., 2010).

TRAF4 is a RING finger domain E3 ubiquitin ligase that is part of the TNF receptor (TNFR)-related junction protein family. Unlike other members of the TRAF family, TRAF4 does not interact directly with TNFR. It is amplified and overexpressed in various cancer types, playing a significant role in promoting cancer progression and metastasis (Ruan et al., 2022). Singh et al. demonstrated that TRAF4 expression is significantly higher in metastatic prostate cancer compared to primary tumors, highlighting its critical role in prostate cancer cell invasion. TrkA, a receptor tyrosine kinase (RTK), binds to nerve growth factor (NGF) at the cell membrane and activates the Ras/MAPK, PI3K, and PLCγ signaling pathways, promoting cell survival, proliferation, and invasion (Molloy et al., 2011). The authors showed that TRAF4 facilitates TrkA ubiquitination through atypical K27 and K29 ubiquitin linkages within its kinase structural domain. This post-translational modification enhances TrkA kinase activity, increases its tyrosine phosphorylation levels, and activates subsequent downstream signaling pathways, thereby promoting prostate cancer metastasis (Singh et al., 2018). Additionally, another study indicated that TRAF4 plays a significant role in desmoplasia-resistant prostate cancer, a condition that presents a major clinical challenge, where the androgen receptor (AR) remains a key oncogenic factor. AR ubiquitination has been shown to modulate AR activity and contribute to the progression of CRPC (Xu et al., 2009). Singh et al. demonstrated that TRAF4 mediates atypical K27-linked AR ubiquitination at its C-terminus. This atypical ubiquitination enhances the interaction between AR and the pioneer transcription factor FOXA1, subsequently altering the AR genome binding profile and promoting the development of CRPC (Singh et al., 2023).

## MDM2, TRIM and F-box proteins in prostate cancer

Mouse double minute 2 (MDM2) has been shown to act as an oncogene in a variety of tumours including prostate, breast, ovarian and liver cancers (Qin et al., 2017; Zheng et al., 2023; Wang W. et al., 2020). It not only serves as a negative regulator of p53 but is also linked to poor prognosis, a high likelihood of recurrence, and resistance to therapy in cancer. Targeted MDM2 therapy is believed to enhance the sensitivity of prostate cancer to androgen deprivation therapy (ADT), either in a p53-dependent or independent manner. Consequently, targeted MDM2 therapy is increasingly recognized as a promising therapeutic strategy in clinical oncology (Feng et al., 2016). Yan et al. discovered that MDM2 directly targets the p53 protein to facilitate its ubiquitylation and degradation, thereby influencing the invasion and migration of PCa cells (Yan et al., 2023). Iron death is an iron-dependent mode of programmed cell death characterized by the accumulation of lipid peroxides, primarily regulated by antioxidant systems such as GPX4 and glutathione. Key regulators include genes such as System Xc- (SLC7A11), the FSP1-CoQ10 pathway, and NRF2—molecules that influence the process of iron death by regulating cystine uptake, lipid metabolism, and oxidative stress. This process holds potential in cancer therapy; for instance, inducing iron death could enhance the anti-cancer effects of photodynamic therapy (Liu et al., 2022; Chang et al., 2024). Alkylglycerol phosphate synthase (AGPS) is a crucial enzyme in ether lipid synthesis that promotes peroxisome formation and increases the susceptibility of tumor cells to iron death (Benjamin et al., 2013). Zhang et al. revealed that MDM2 promotes the ubiquitination and degradation of AGPS in a p53-independent manner. Additionally, TrkA enhances the degradation of AGPS and can regulate the progression of PCa by modifying the phosphorylation of AGPS, which amplifies the function of MDM2 in the ubiquitination and degradation of AGPS. TrkA inhibitors have the potential to protect AGPS and promote iron death in prostate cancer, exhibiting improved anticancer efficacy when combined with iron death inducers (Zhang et al., 2024).

The tripartite motif (TRIM) family constitutes the largest subfamily of proteins that contain RING structural domains. In addition to an N-terminal RING structural domain, these proteins possess one or two additional zinc-binding B-box structural domains (B-box 1 and B-box 2) and a complex helical region, thus giving rise to the acronym TRIM, or RBCC, which denotes both the module and the entire family (Reymond et al., 2001). The RING structural domain confers E3 ubiquitin ligase activity to TRIM family members within the ubiquitination cascade (Meroni and Diez-Roux, 2005; Komander and Rape, 2012). TRIM36, a novel androgen-responsive gene, has been shown to regulate tumor plasticity in neuroendocrine prostate cancer (NEPC) by repressing glutathione peroxidase 4 (GPx4) expression in a manner dependent on HK2 ubiquitination. The proposed mechanism involves the upregulation of TRIM36 during androgen deprivation therapy (ADT), which enhances the ubiquitination of HK2 linked to lysine 48, thereby inhibiting glycolysis. Furthermore, depletion of HK2 leads to a reduction in GPx4 expression, ultimately promoting ferroptosis. The inhibition of glycolysis and activation of ferroptosis are integral to neuroendocrine differentiation (NED) in PCa, and these effects can be reversed by sh-TRIM36. The reduction of GPx4 expression by TRIM36 *via* HK2 ubiquitination represents a crucial mechanism for regulating NEPC (Zhao et al., 2023). Transmembrane tetraspanins (TSPAN) are small transmembrane glycoproteins characterized by four highly conserved transmembrane structural domains, and they are expressed across a broad range of organisms. Research has demonstrated that TSPAN18 can directly interact with STIM1, protecting it from E3 ligase TRIM32-mediated ubiquitination. Additionally, TSPAN18 has been shown to promote PCa cell metastasis by modulating the STIM1-dependent Ca2+ signaling pathway, thereby further elucidating the positive role of TSPAN18 in regulation-mediated carcinogenesis (Zhou et al., 2023). SERPINH1 was initially identified as an endoplasmic reticulum retention protein, and recent studies have established a connection between aberrant SERPINH1 expression and tumorigenesis in malignant tumors (Xiong et al., 2020). It has been reported that SERPINH1 is upregulated in PCa bone metastasis, where it induces PCa cell bone metastasis *in vivo*, promotes cellular proliferation, and attenuates apoptosis. This study demonstrates that SERPINH1 binds to P62, reduces TRIM21-mediated degradation of K63-linked P62 ubiquitination, and enhances PCa proliferation and resistance to apoptosis. Furthermore, SERPINH1 regulates the ubiquitination and degradation of P62, thereby promoting PCa bone metastasis, which may be considered a potential target for the treatment of metastatic PCa (Tang et al., 2024).

The Cullin-RING ligase complex family includes Skp1-Cullin1-F-box protein (SCF)-type ligases, which are composed of Skp1, Cullin1 (Cul1), Rbx1, and F-box proteins, and represents one of the large E3 enzyme families (Lin et al., 2019). F-box proteins, known as subunits of the SCF E3 ligase complex, are commonly categorized into three subfamilies: FBXW (F-box with WD 40 amino acid repeats), FBXL (F-box with leucine-rich amino acid repeats), and FBXO (F-box with uncharacterized structural domains). F-box proteins have been implicated in the development of various diseases, including cancer (Yan et al., 2020). Zhou et al. discovered that F-box and WD repeat structural domain 2 (FBXW2) is downregulated in highly metastatic PCa cells and tissues. They found that FBXW2 binds to the epidermal growth factor receptor (EGFR) at its shared degron motif (TSNNST), promoting EGFR ubiquitination and degradation. Furthermore, overexpression of FBXW2 in both *in vitro* and *in vivo* PCa models resulted in reduced growth and metastasis, whereas depletion of FBXW2 produced the opposite effect. Thus, FBXW2 inhibits PCa cell proliferation and metastasis by targeting EGFR for ubiquitination and degradation, thereby suppressing downstream EGFR signaling (Zhou et al., 2022). NDR1 is a crucial kinase in the HIPPO pathway, involved in the regulation of cell proliferation, apoptosis, and the maintenance of tissue morphology (Century et al., 1997). It has been demonstrated that NDR1 phosphorylates β-catenin at the Ser33/37 locus, which enhances its interaction with FBXO11. This interaction facilitates the FBXO11-mediated ubiquitination and subsequent cytoplasmic degradation of β-catenin, while the NDR1-FBXO11 complex inhibits β-catenin’s nuclear translocation by promoting JNK2 ubiquitination. Consequently, NDR1 and FBXO11 collaboratively regulate β-catenin activity in prostate cancer cells through a mechanism of dual phosphorylation-driven ubiquitination, potentially suppressing EMT (Xuan et al., 2024). The miR-30 family functions as tumor suppressors in various cancers, including PCa (Kao et al., 2014). Studies have indicated that miR-30e is downregulated in PCa cells and is implicated in the regulation of the cell cycle, apoptosis, and drug sensitivity. Furthermore, miR-30e has been shown to interact with the mRNAs of AR, FBXO45, SRSF7, and MYBL2, thereby altering their expression in PCa cells. Notably, in the case of miR-30e’s interaction with FBXO45, FBXO45 mRNA is not expressed in PCa cells. In this context, FBXO45 mRNA is the target of miR-30e; FBXO45 directly targets and degrades p73, while the knockdown of FBXO45 results in the stabilization of p73 and induces apoptosis in a p53-independent manner (Ganapathy et al., 2022).

## Other ubiquitin enzymes in prostate cancer

The androgen receptor plays a crucial role in all stages of prostate carcinogenesis, including the progression of PCa (Chan and Dehm, 2014). It comprises an N-terminal structural domain (NTD), a DNA-binding structural domain (DBD), a hinge region, and a ligand-binding structural domain (LBD) (Gel and mann, 2002). Downregulation of the AR remains an effective treatment strategy for PCa, even during the phase of depot resistance (Fletcher et al., 2019). The aryl hydrocarbon receptor (AHR) is highly expressed in various organs and tissues, and there is increasing evidence that AHR plays a significant role in cellular homeostasis and disease (Hahn et al., 2009). Studies have demonstrated that AHR functions as an E3 ubiquitin ligase, mediating the ubiquitination and degradation of AR in prostate cancer (Chen et al., 2020). Siah2, a RING-finger-type ubiquitin ligase characterized by its N-terminal catalytic RING domain, two zinc fingers, and a C-terminal substrate-binding domain (SBD), has recently been identified as an E3 ubiquitin ligase for AR. This ligase specifically targets NCOR1-binding, inhibitory, and cytokine-conjugating enzymes for degradation. Furthermore, Siah2 is involved in lipid metabolism, cell motility, and the proliferation of prostate cancer cells (Jing et al., 2018). Forkhead box protein A1 (FOXA1) acts as a transcriptional activator of steroid hormone receptors, including AR. In CRPC, the nuclear localization and overexpression of FOXA1 enhance tumor growth and metastasis by facilitating cell cycle progression (Jain et al., 2011). SKP2 is a substrate-recognizing component of the SCF E3 ubiquitin ligase complex. Celada et al. demonstrated a direct interaction between SKP2 and FOXA1 proteins in PCa. FOXA1 serves as a substrate for SKP2-mediated ubiquitination *via* the K6 and K29 linkages. Through this pathway, SKP2 catalyzes the nonclassical ubiquitination of FOXA1, leading to its lysosome-dependent degradation, which promotes the ontogeny and progression of PCa (Celada et al., 2023).

Approximately 75% of the human genome is transcribed into RNA, with only 3% being translated into protein-coding mRNAs. Non-coding RNAs (ncRNAs) are classified into various categories based on their length, shape, and location. Numerous studies have underscored the significant role of microRNAs (miRNAs) in various cancers, where many miRNAs are found to be highly expressed in cancer cells, thereby promoting cancer development. Similar to miRNAs, long non-coding RNAs (lncRNAs) also function as oncogenes or tumor suppressors, influencing tumorigenesis and progression (Yan and Bu, 2021). In their research, Dallavalle et al. established a connection between miRNA dysregulation and altered protein ubiquitination, revealing that miR-424 is upregulated in prostate tumors and correlates with invasive features. They demonstrated that the E3 ubiquitin ligase COP1 interacts with STAT3, mediating its ubiquitination and degradation, while miR-424 promotes STAT3 stabilization and activity, thereby facilitating tumor progression by targeting COP1 (Dallavalle et al., 2016). Additionally, lncRNAs play a role in the ubiquitination process, as highlighted in another study on prostate cancer, which found that the lncRNA MIAT is highly expressed in prostate adenocarcinoma (PRAD) tissues and is associated with poor prognosis. Inhibition of MIAT was shown to suppress the malignant biological behaviors of PRAD cells, while depletion of MIAT enhanced the immune response of CD8^+^ T cells and hindered the immune escape of PRAD cells. Further investigations revealed that MIAT downregulates TP53 protein expression through ubiquitin modification by recruiting the transducin β-like protein 1X (TBL1X). Silencing TP53 or overexpressing TBL1X was sufficient to diminish the tumor suppressor effects of MIAT knockdown in both *in vitro* and *in vivo* models (Gong et al., 2023). The ASB family of proteins comprises a group of E3 ubiquitin ligases characterized by an N-terminal anchor protein repeat domain that facilitates substrate recognition, as well as a C-terminal suppressor of cytokine signaling (SOCS) box that plays a role in protein ubiquitination (Liu et al., 2019). ASB1 is a key member of the ASB family and one study analysed expression levels in tumour tissue and adjacent paracancerous tissue using data from TCGA and GTEx. Their analysis showed that ASB1 expression was significantly downregulated in prostate cancer tissues compared to paraneoplastic tissues, which was further confirmed by qRT-PCR experiments, and that silencing of ASB1 promotes prostate cancer cell proliferation *in vitro*. This investigation further elucidated that ASB1 interacts with CHCHD3 and enhances its K48-linked ubiquitination, thereby influencing the behavior of prostate cancer cells (Zhao et al., 2024). UBE2N (also known as Ubc13) is an E2 ubiquitin-conjugating enzyme responsible for the synthesis of lysine 63-linked polyubiquitin chains (Cheng et al., 2014). Recent findings indicate that the upregulation of UBE2N correlates with poor prognosis in prostate cancer, and that the knockdown of UBE2N inhibits cell viability and glycolysis in prostate cancer cells. Additionally, UBE2N promotes the ubiquitination and degradation of Axin1, and the overexpression of Axin1 negates the effects of UBE2N on the viability and glycolysis of prostate cancer cells (Yang et al., 2024).

## The role of ubiquitination in bladder cancer

Long-stranded noncoding RNA small nucleolar RNA host gene 1 (lncRNA SNHG1) is well-known for its association with tumor stage, size, and overall survival (Thin et al., 2019). The overexpression of MDM2 has been reported to counteract the inhibitory effects of miR-379–5p on the proliferation, migration, and invasive capabilities of bladder cancer cells (Wu D. et al., 2017). In the presence of EGFR, MDM2 binds to peroxisome proliferator-activated receptor-γ (PPARγ) and modulates the ubiquitination of the PPARγ protein in colon cancer cells (Xu et al., 2016). Cai et al. observed that bladder cancer patients with high SNHG1 expression exhibited poorer prognoses. They further demonstrated that SNHG1 promotes the proliferation of bladder cancer cells by inhibiting apoptosis, thereby corroborating their hypothesis that SNHG1 can inhibit apoptosis. Additionally, they found that SNHG1 enhances the proliferation of bladder cancer cells through the suppression of apoptosis and promotes MDM2 expression by binding to miR-9-3p, which in turn facilitates the ubiquitination and downregulation of PPARγ. This cascade of events leads to increased proliferation of bladder cancer cells *in vitro* and contributes to tumorigenesis *in vivo* (Table 2) (Cai et al., 2022). Another study reported that lncRNA and MDM2 influence cancer progression through ubiquitination in bladder cancer, particularly investigating the mechanism by which lncRNA plasmacytoma variant translocation 1 (PVT1) regulates adriamycin (ADM) resistance in bladder cancer cells. This study demonstrates that PVT1 interacts with and enhances MDM2 expression, leading to the upregulation of MDM2-mediated aurora kinase B (AURKB) activity, which mechanistically increases the ubiquitination of p53 by MDM2. Collectively, these findings indicate that PVT1 promotes breast cancer cell proliferation and drug resistance through the upregulation of MDM2 and AURKB-mediated p53 ubiquitination (Jiang et al., 2022). Conversely, circPKN2, another non-coding RNA, has been shown to inhibit BC cell proliferation and migration *in vitro*. The proposed mechanism suggests that circPKN2 recruits STUB1, thereby facilitating the ubiquitination of SCD1, which inhibits the WNT pathway and promotes ferroptosis in BC. Additionally, the study reveals a regulatory role for the splicing factor QKI in the biogenesis of circPKN2. Animal studies further demonstrate that circPKN2 enhances ferroptosis in BC cells *in vivo*, while inhibiting tumor growth and metastasis (Liu et al., 2024).

**TABLE 2:** Ubiquitination enzymes regulate the development and progression of bladder cancer.

E2s/E3s	Starting factor	Pro/Anti tumor	Ubiquitination substrate	Biochemical function	Outcomes	References
E3	MDM2	Pro-tumor	PPARγ		Elevated proliferation of bladder cancer cells *in vitro* and tumorigenesis *in vivo*	Cai et al. (2022)
E3	MDM2	Pro-tumor	p53		Promotes BC cell proliferation and drug resistance	Jiang et al. (2022)
E3	STUB1	Anti-tumor	SCD1		Enhancement of iron death in BC cells and inhibition of tumor growth and metastasis	Liu et al. (2024)
E3	RNF26	Pro-tumor	TRIM21	K48 polyubiquitin chain	Induction of bladder cancer cell proliferation and migration	Yao et al. (2024)
E3	TRIM65	Pro-tumor	ANXA2		Increased invasiveness of UCB cells	Wei et al. (2018)
E3	FBXW7	Pro-tumor	ZMYND8		Promote Bca growth and migration	Qiu et al. (2021)
E2	UBE2S	Pro-tumor	LPP	K11 polyubiquitin chain	Promotes EMT and enhances lymphatic transfer of BCa	Xiao et al. (2023)

ER-embedded RING finger protein 26 (RNF26) is a critical E3 ubiquitin ligase that mediates the ubiquitination of spirochete ubiquitination 1 (SQSTM1) through its interaction with the ubiquitin-conjugating enzyme UBE2J1. This interaction facilitates the anchoring of homologous vesicles to the perinuclear region of the cell and promotes the termination of EGF-induced AKT signaling (Cremer et al., 2021). Furthermore, RNF26 has been shown to interact with another E3 ligase, TRIM21, enhancing its K48-linked ubiquitination in bladder cancer cells. In this context, TRIM21 functions as an E3 ligase that mediates the ubiquitination and subsequent degradation of ZHX3. The RNF26/TRIM21 complex is implicated in the proliferation and migration of bladder cancer cells *via* ZHX3 (Yao et al., 2024). Additionally, TRIM65 was identified as a significant oncogenic factor in urothelial carcinoma of the bladder (UCB) through screening of The Cancer Genome Atlas (TCGA) database and was validated in numerous clinical UCB tissue samples by Wei et al. Their study revealed that TRIM65 regulates cytoskeletal rearrangement and induces epithelial-mesenchymal transition in UCB cells through the ubiquitination of ANXA2, which ultimately enhances the invasiveness of these cells (Wei et al., 2018). ZMYND8 is an epigenetic regulator that has been identified as a common oncogene in various tumors. Qiu et al. first reported that the level of ZMYND8 protein was significantly elevated in BCa samples compared to normal tissues. Their predictive bioinformatics analysis indicated that the E3 ubiquitin ligase FBXW7 directly interacts with ZMYND8 and facilitates its degradation through a polyubiquitylation mechanism. Low levels of FBXW7 are identified as a risk factor that promotes and relies on the accumulation of ZMYND8 protein, thereby facilitating the growth and migration of BCa (Qiu et al., 2021). Additionally, UBE2S, as part of the ubiquitin-proteasome system (UPS), has been shown to promote tumor development by either degrading or stabilizing various proteins. Notably, UBE2S expression is strongly correlated with lymphatic metastasis and serves as an independent prognostic factor in BCa patients. In this study, UBE2S knockdown inhibited BCa migration and invasion *in vitro*, as well as lymphatic metastasis *in vivo*. Mechanistically, UBE2S interacts with TRIM21 to induce the ubiquitination of lipoma preferred partner (LPP) *via* K11 linkage, with LPP acting to inhibit the pro-metastatic effects of UBE2S on BCa (Xiao et al., 2023).

## The role of ubiquitination in kidney disease

### Ubiquitination in kidney cancer

Large tumor suppressor 1 (LATS1) is a serine/threonine kinase belonging to the AGC kinase family and has been identified as a tumor suppressor that is downregulated in various types of human cancers (Visser and Yang, 2010). In clear cell renal cell carcinomas, SPOP is consistently overexpressed and accumulates in the cytoplasm of ccRCC cells, in contrast to its predominant nuclear localization in other cell types (Liu et al., 2009). SPOP has been identified as a novel E3 ligase for LATS1 in ccRCC cells, with the E3 ubiquitin ligase Cullin3/SPOP mediating the stability of LATS1 through polyubiquitination, leading to its degradation in a degron-dependent manner in renal carcinoma. This study collectively demonstrates that SPOP promotes the ubiquitination and degradation of LATS1, thereby enhancing renal cancer cell invasion (Table 3) (Wang L. et al., 2020). Additionally, serine/threonine protein phosphatase-5 (PP5), which belongs to the phosphoprotein phosphatase (PPP) family, is unique in that it is encoded by a single gene, with its regulatory and catalytic domains contained within the same polypeptide. PP5 plays a crucial role in regulating hormone- and stress-induced signaling networks that enable cells to respond appropriately to genomic stresses (Golden et al., 2008). Through mass spectrometry (MS) analysis, Dushukyan et al. identified VHL as a binding partner for PP5, revealing that the VHL E3 ligase ubiquitinates and degrades PP5 in the proteasome. They further discovered that the VHL E3 ligase targets the K185 and K199 residues in PP5 for ubiquitination, and that the downregulation of PP5 leads to apoptosis in VHL-deficient ccRCC cells (Dushukyan et al., 2017).

**TABLE 3:** Ubiquitination enzymes regulate the development and progression of kidney disease.

Kidney diseases	E2s/E3s	Starting factor	Pro/Anti tumor	Ubiquitination substrate	Outcomes	References
kidney cancer	E3	SPOP	Pro-tumor	LATS1	Enhancement of renal cancer cell invasion	Wang et al. (2020c)
	E3	VHL	Anti-tumor	PP5	Influence on ccRCC apoptosis	Dushukyan et al. (2017)
kidney injury	E3	HRD1		GSK3β、CK1α	Promoting acute kidney injury	Sun et al. (2022)
	E3	HRD1		NRF2	Protecting the kidney from ischemia-reperfusion injury	Zhang et al. (2021a)
	E3	WWP2		p53	Promotes proliferation and inhibits apoptosis of renal tubular epithelial cells	Che et al. (2020)
	E3	STUB1		CFTR	Exacerbation of calcium oxalate-induced renal injury	Hou et al. (2024)
renal fibrosis	E3	Smurf2		SnoN、Ski、TGIF	Dysregulation of Smurf2 expression affects pro-fibrosis	Tan et al. (2008)
chronic kidney disease	E2	UBE2O		Bcl-xL	Shortened platelet lifespan, ultimately leading to decreased platelet counts	Lan et al. (2022)

## Ubiquitination in kidney injury

The pathogenesis of acute kidney injury (AKI) is linked to the activation of several signaling pathways, notably Wnt/β-catenin signaling. The activation of the Wnt/β-catenin pathway is crucial in the context of severe AKI (Lim and Nusse, 2013). S100 calcium-binding protein A16 (S100A16), a novel member of the S100 family of calcium-binding proteins, serves as a multifunctional signaling factor implicated in various pathogenic mechanisms, including tumors, disturbances in glucose and lipid metabolism, and chronic kidney disease (CKD) (Heizmann et al., 2002). Research has demonstrated that S100A16 knockdown mitigates renal injury in mouse models of AKI, and it has been shown to reduce the activation of the Wnt/β-catenin pathway in these models. Further mechanistic studies indicate that S100A16 regulates HRD1 in the activation of the Wnt/β-catenin pathway by physically binding to and promoting the ubiquitination of GSK3β and CK1α (Sun et al., 2022). Additionally, another study reported that HRD1 is involved in kidney injury through ubiquitination, highlighting that X-box binding protein 1 (XBP1) and its downstream target HRD1 are engaged in AKI by modulating the NRF2/HO-1-mediated response to oxidative stress, which is recognized as a critical factor in kidney injury. This research elucidates the involvement of XBP1 and HRD1 in the interplay between endoplasmic reticulum stress (ERS) and mitochondrial dysfunction *via* the regulation of the NRF2/HO-1-mediated response to reactive oxygen species (ROS) signaling. Downregulation of XBP1 in renal epithelial cells results in decreased HRD1 expression and enhanced NRF2/HO-1 function. HRD1, an E3 ligase, facilitates NRF2 downregulation *via* the ubiquitination degradation pathway. The downregulation of XBP1 protects the kidney from ischemia-reperfusion injury by inhibiting HRD1-mediated ubiquitination of NRF2 (Zhang J. et al., 2021). Another investigation into ischemia-reperfusion revealed an association between the E3 ubiquitin ligase gene WWP2 and acute AKI. This study demonstrated that WWP2 was downregulated while p53 was upregulated in ischemia-reperfusion (IR)-induced HK-2 cells. Furthermore, WWP2 overexpression promoted proliferation and inhibited apoptosis in human renal proximal tubular epithelial cells (HK-2), indicating a protective role against AKI through the mediation of p53 ubiquitination and degradation (Che et al., 2020)。Calcium oxalate (CaOx) crystals, which form in the kidney, can cause renal epithelial damage and contribute to the progression of crystalline nephropathy. Bioinformatics analysis has predicted the ubiquitination binding site of the cystic fibrosis transmembrane conductance regulator (CFTR) to U-box protein 1 (STUB1), thereby confirming STUB1’s role as a ubiquitin ligase in CFTR degradation. Knockdown of STUB1 resulted in upregulation of CFTR expression, while STUB1 overexpression produced the opposite effect. Additionally, knockdown of CFTR negated the impact of STUB1 deficiency on autophagy. *In vivo* experiments indicated that CFTR overexpression mitigated renal tissue injury and CaOx deposition in mice, with STUB1-mediated ubiquitination of CFTR playing a crucial role in alleviating calcium oxalate-associated renal injury by regulating autophagy (Hou et al., 2024).

## Ubiquitination in renal fibrosis and chronic kidney disease

TGF-β plays a crucial role in regulating various biological processes, including cell proliferation, apoptosis, differentiation, and extracellular matrix production. Upon the binding of TGF-β to its type II serine/threonine kinase receptor (TβRII), TβRI is activated, which leads to the phosphorylation and activation of downstream receptor-regulated Smads (R-Smads) (Böttinger and Bitzer, 2002; Feng and Derynck, 2005). Recent findings indicate that Smurf2 is induced in the renal tubules of human fibrotic kidneys. *In vitro* studies have shown that TGF-β1 induces Smurf2 expression in renal tubular cells; moreover, the overexpression of the Smad co-repressor protein SnoN completely abolishes TGF-β1-mediated Smurf2 mRNA induction in HKC-8 cells. The E3 ligase Smurf2 specifically downregulates Smad2, as well as SnoN, Ski, and TGIF in renal tubular epithelial cells, thereby influencing the progression of renal fibrosis (Tan et al., 2008). Thrombosis and hemorrhage represent two opposing pathologies that are prevalent in the CKD population. Platelet homeostasis is central to the pathogenesis of these conditions, which varies among individuals with CKD, although the underlying mechanisms remain poorly understood. Lan et al. demonstrated that platelet counts are reduced in both patients with advanced CKD (Adv-CKD) and in a corresponding mouse model, with a positive correlation observed between platelet counts and circulating Klotho levels. They identified that the ubiquitin ligase UBE2O regulates the ubiquitination and degradation of Bcl-xL in platelets. Furthermore, oxidative stress induced by Adv-CKD in platelets activates p38MAPK, which promotes the phosphorylation of Bcl-xL. This phosphorylation enhances the binding of UBE2O to Bcl-xL, leading to its subsequent degradation. Consequently, the lifespan of platelets is diminished in CKD patients, resulting in decreased platelet counts (Lan et al., 2022).

## The role of deubiquitination in urologic diseases

### Deubiquitination in prostate cancer

Persistent aberrant activation of the AR signaling pathway is a significant factor in the progression of CRPC. Currently, one of the primary therapeutic strategies for CRPC involves tumor suppression by targeting the AR, with enzalutamide being a prominent example. However, CRPC tumor cells often develop resistance to drugs such as enzalutamide through mechanisms including AR point mutations, AR amplification, alterations in androgen biosynthesis, and other factors (Fujita and Nonomura, 2019). As research advances, it has become evident that the regulation of the AR signaling pathway in CRPC is influenced by various elements, including LncRNAs that have recently been implicated in malignant tumors. Zhang et al. reported that the LncRNA PCBP1-AS1 was significantly upregulated in CRPC. Targeting PCBP1-AS1 *in vitro* markedly inhibited the proliferation and migration of CRPC cell lines. Furthermore, *in vivo* inhibition of PCBP1-AS1 significantly suppressed tumor growth. Investigating the underlying mechanism, they discovered that PCBP1-AS1 stabilized the USP22-AR/AR-V7 complex, enhanced the deubiquitination of AR/AR-V7, and prevented the protein from being degraded *via* the ubiquitin-proteasome pathway by binding to the NTD of AR/AR-V7 (Table 4) (Zhang B. et al., 2021). Additionally, another study demonstrated that USP22 modulates both the androgen receptor and Myc to drive AR-driven cancer cell proliferation and tumor growth in CRPC cells (Nag and Dutta, 2020). The PD-L1/PD-1 signaling pathway is a crucial component of tumor immunosuppression; however, the expression of PD-L1 is regulated by complex mechanisms, including gene transcription as well as post-transcriptional and post-translational modifications. Evidence indicates that the expression of PD-L1 protein is often regulated through the proteasomal degradation pathway (Lim et al., 2016). Fu et al. demonstrated that NDR1 enhances the level of PD-L1 expression in PCa and facilitates immune evasion by tumors. They employed mass spectrometry to identify and analyze USP10, a deubiquitinating enzyme associated with NDR1 that stabilizes PD-L1 (Fu et al., 2024). Additionally, insulin-like growth factor (IGF) mediates various biological activities, such as growth, anti-apoptosis, and differentiation in numerous cell types. The biological effects of IGF are primarily mediated by insulin receptor substrates (IRS)-1 and IRS-2 (Jones and Clemmons, 1995). The deubiquitinase, ubiquitin-specific peptidase 9X (USP9X), has been identified as a novel binding partner for IRS-2. In a human prostate cancer cell line, small interfering RNA (siRNA)-mediated knockdown of USP9X resulted in decreased levels of IGF-IR and IRS-2 proteins, along with increased ubiquitination of these proteins. The knockdown of USP9X inhibited the basal activation of the Erk1/2 pathway, while ectopic expression of IRS-2 significantly restored this pathway; however, IGF-IR did not have the same effect. This suggests that the stabilization of IRS-2 by USP9X is essential for the basal activation of Erk1/2 (Furuta et al., 2018). The NADPH oxidase (Nox) family of enzymes specializes in the production of ROS. ROS generated by Nox are implicated in various signaling cascades and pathophysiological conditions, including cancer. A genome-wide screen for deubiquitinating enzymes that regulate Nox organizer 1 (NoxO1) protein expression was conducted using a CRISPR/Cas9-mediated DUB knockdown library. This screen identified cylindromatosis (CYLD) as a binding chaperone that regulates NoxO1 protein expression. It was demonstrated that the overexpression of CYLD promotes the ubiquitination of NoxO1 and shortens its half-life. Furthermore, CRISPR/Cas9-mediated knockdown of CYLD in PC-3 cells enhanced cell proliferation, migration, colony formation, and invasion *in vitro* (Haq et al., 2022).

**TABLE 4:** Deubiquitination enzymes regulate the development and progression of urologic diseases.

urologic diseases	Starting factor	Pro/Anti tumor	Promotes OR inhibition of substrate deubiquitination	Outcomes	References
prostate cancer	USP22	Pro-tumor	Promotes AR/AR-V7 deubiquitination	Promoting proliferation and migration	Zhang et al. (2021b)
	USP22	Pro-tumor	Promotes AR, Myc deubiquitination	Driving cancer cell proliferation and tumor growth	Nag and Dutta (2020)
	USP10	Pro-tumor	Promotes PD-L1 deubiquitination	Promoting immune escape from tumors	Fu et al. (2024)
	USP9X	Pro-tumor	Promotes IRS-2 deubiquitination	Promote prostate cancer growth	Furuta et al. (2018)
	CYLD	Pro-tumor	Inhibits NoxO1 deubiquitination	Promoted cell proliferation, migration, colony formation and invasion *in vitro*	Haq et al. (2022)
bladder cancer	USP21	Pro-tumor	Promotes EZH2 deubiquitination	Promote BC cell transfer	Chen et al. (2017)
	USP21	Pro-tumor	Promotes p65 deubiquitination	Promotes bladder cancer cell growth, EMT and metastasis	Ma et al. (2024)
	OTUB1	Pro-tumor	Promotes E2F1 deubiquitination	Promotes BC cell proliferation, migration and invasion and inhibits apoptosis	Zhang et al. (2021c)
kidney cancer	USP37	Pro-tumor	Promotes HIF2α deubiquitination	USP37 depletion leads to reduced primary renal tumorigenesis and spontaneous lung metastases	Hong et al. (2020)
	USP13	Pro-tumor	Promotes ZHX2 deubiquitination	USP13 Promoting Cell Proliferation	Xie et al. (2022)

## Deubiquitination in bladder cancer

Ubiquitin-specific protease 21 (USP21) is a member of the USP family of DUB characterized by a C-terminal catalytic DUB structural domain. USP21 is believed to catalyze the hydrolysis of ubiquitinated histone H2A (ubH2A) and facilitate transcriptional initiation (Nakagawa et al., 2008). A study conducted by Chen et al. demonstrated that USP21 is highly expressed in BC, with its expression correlating to tumor size, metastasis, and poor survival outcomes. They further established that USP21 promotes BC cell metastasis by deubiquitinating and stabilizing EZH2 (Chen et al., 2017). Additional insights into the role of USP21 in bladder cancer were provided by Ma et al., who identified USP21 as a deubiquitinase for p65 that prevents the degradation of the K48-linked ubiquitin chain on p65, thereby promoting the growth, EMT, and metastasis of bladder cancer cells through its deubiquitinating activity. Notably, 20-hydroxyecdysone (20-HE) can directly inhibit NF-κB/p65 signaling at the transcriptional level while also serving as an inhibitor of USP21, leading to p65 protein degradation and the blockade of its activation, ultimately preventing the progression of bladder cancer (Ma et al., 2024). Recent studies have shown that TMPO antisense RNA 1 (TMPO-AS1) functions as a competing endogenous RNA by forming a sponge for microRNA (miRNA) in various cancers. A new study further demonstrates that TMPO-AS1 is upregulated in BC tissues, where it promotes BC cell proliferation, migration, invasion, and survival *in vitro*. OTUB1 is a founding member of the OTU structural domain deubiquitinase family, classified as cysteine proteases. It is highly expressed in several organs, including the kidney, spleen, and prostate (Balakirev et al., 2003). The enzyme specifically recognizes Lys48-linked polyubiquitin chains, and its catalytic activity is reliant on the classical catalytic triad composed of Cys91, His265, and Asp267 (Zhu et al., 2021). Structural analysis has revealed that the unique N-terminal alpha helix of OTUB1 plays a critical role in substrate binding and catalytic regulation by making direct contact with proximal ubiquitin molecules (Mevissen et al., 2013). E2F1, a member of the E2F transcription factor family, which consists of eight proteins, acts as a transcriptional activator. This study reveals that TMPO-AS1 regulates the protein level of E2F1 through protein stabilization, specifically by enhancing E2F1 stability *via* OTUB1-mediated deubiquitylation. Additionally, E2F1 promotes the proliferation, migration, and invasion of BC cells while inhibiting apoptosis *in vitro*. Overall, TMPO-AS1 regulates the malignant phenotype of BC cells through its interaction with E2F1 (Zhang Y. et al., 2021).

## Deubiquitination in kidney cancer

Von Hippel-Lindau (VHL) is the most significant oncogene in kidney cancer, being lost or mutated in over 70% of cases. Research conducted across various laboratories has demonstrated that the pVHL-associated complex possesses E3 ubiquitin ligase activity. The loss of VHL results in the accumulation of hypoxia-inducible factor α (HIF-α), which includes HIF1α and HIF2α, along with other potential substrates such as ZHX2 and SFMBT1 (Kaelin, 2002). HIF2α, a crucial subunit of HIF, has been shown to facilitate renal carcinogenesis and the progression of renal cancers both *in vitro* and *in vivo* (Raval et al., 2005). Hong et al. performed a deubiquitinase complementary DNA (cDNA) library binding screen, revealing that ubiquitin-specific peptidase 37 (USP37) is a DUB that binds to and enhances the deubiquitination of HIF2α. Consequently, USP37 promotes the stability of HIF2α in an enzyme-dependent manner, and the depletion of USP37 results in the downregulation of HIF2α in ccRCC, leading to reduced primary renal tumorigenesis and spontaneous lung metastasis (Hong et al., 2020). Conversely, recently developed HIF2α inhibitors have been shown to inhibit tumor growth in certain preclinical renal cancer models, but not in others (Chen et al., 2016). Therefore, targeting factors within the HIF2α-independent signaling pathway in ccRCC, such as ZHX2, may prove to be crucial. Xie et al. demonstrated that ZHX2 promotes ccRCC tumorigenesis independently of HIF. They conducted a cDNA library binding screen and identified USP13 as a DUB that interacts with ZHX2 and facilitates its deubiquitination. USP13 enhances ZHX2 protein stability in an enzyme-dependent manner, and its depletion results in ZHX2 downregulation in ccRCC. Furthermore, USP13 is essential for ccRCC tumor growth *in vivo*, and its effects are partially mediated through its regulation of ZHX2 (Xie et al., 2022).

## Targeted ubiquitination regulates urologic diseases

**FIGURE 2:**
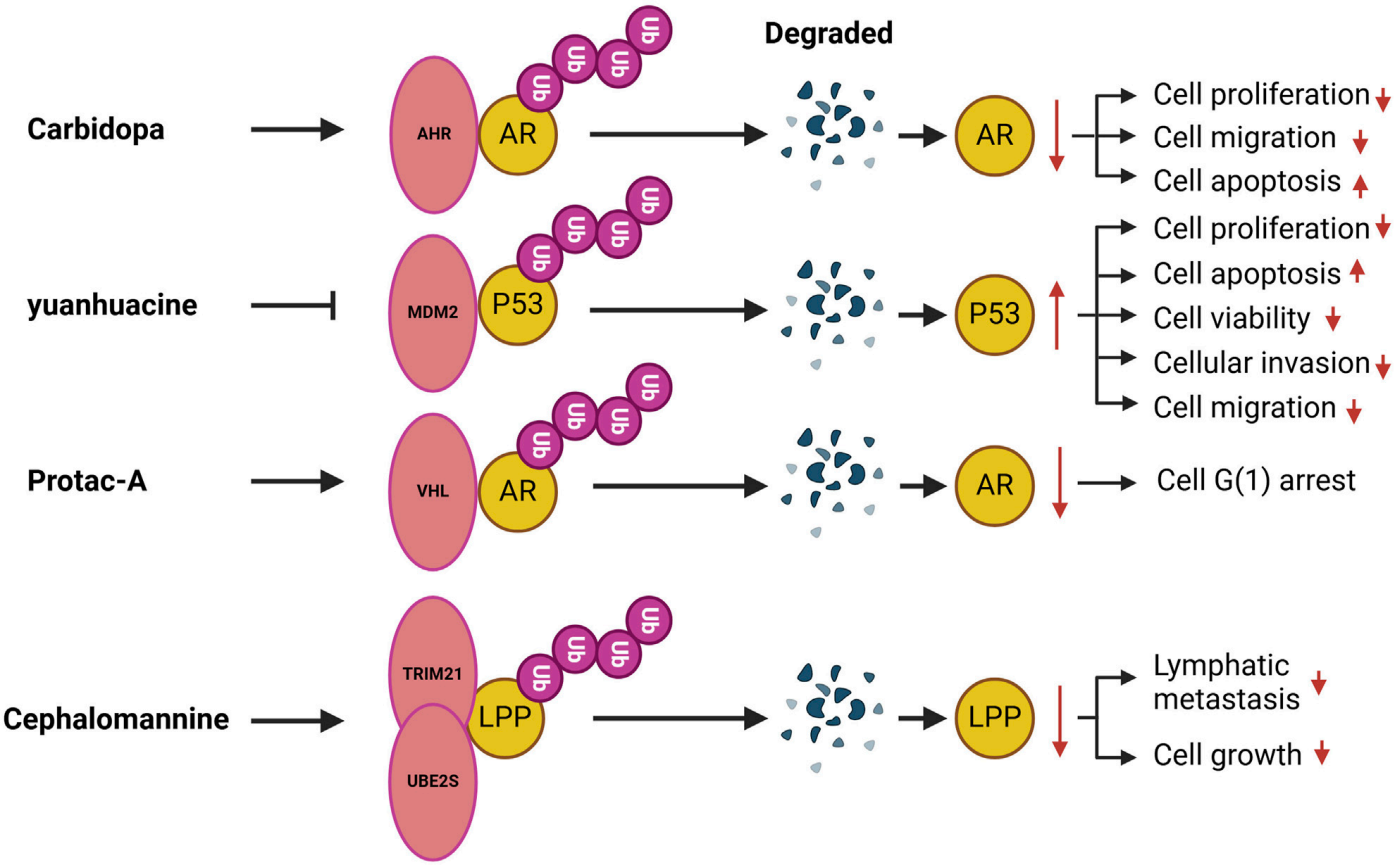
Compounds target ubiquitination in urological disorders. AHR, Aromatic hydrocarbon receptor; MDM2, Mouse double minute 2; VHL, Von Hippel Lindau; TRIM, The tripartite motif; LPP, Lipoma preferred partner.

A number of compounds have been identified that target ubiquitination and modulate urological disorders (Figure 2).Carbidopa, a peripheral decarboxylase inhibitor commonly used in conjunction with L-DOPA for the treatment of Parkinson’s disease (Seeberger and Hauser, 2009), has garnered significant interest in recent years due to its potential anticancer effects. These effects may be associated with the activation of the AHR, which plays a crucial role in maintaining cellular homeostasis. AHR is overexpressed in various tumors, including pancreatic cancer, suggesting that it could serve as an important drug target for certain tumor types (Koliopanos et al., 2002). Research has demonstrated that Carbidopa inhibits PCa growth *in vivo* and reduces AR protein levels through AHR-induced proteasomal degradation. Additionally, Carbidopa treatment has been shown to increase AHR protein levels while decreasing AR protein levels in tumor tissue (Chen et al., 2020). Yuanhuacine, an active ingredient isolated from Daphne genkwa, has demonstrated efficacy in inhibiting tumorigenesis across various cancers. Studies indicate that yuanhuacine inhibits PCa cell proliferation in a dose-dependent manner while also inducing apoptosis. Furthermore, yuanhuacine has been shown to impede PCa cell invasion and migration. Mechanistically, yuanhuacine reduces the ubiquitination and degradation of the p53 protein, leading to an increase in p53 levels. This effect is regulated by the inhibition of both the phosphorylation and total protein levels of MDM2 in murine models. Additionally, yuanhuacine has been found to inhibit the expression of LINC00665, and the upregulation of LINC00665 counteracts the yuanhuacine-mediated inhibition of MDM2 protein expression, thereby suppressing p53 levels through enhanced ubiquitination in yuanhuacine-treated cells (Yan et al., 2023). Protein hydrolysis-targeted chimeric molecules, known as Protacs, exploit the ubiquitin-dependent protein hydrolysis system of eukaryotic cells to target proteins for degradation. Notably, studies have devised Protac-A, which incorporates a peptide ‘degron’ from hypoxia-inducible factor 1alpha to bind to the VHL E3 ubiquitin ligase complex. Treatment with Protac-A in androgen-dependent prostate cancer cells results in G1 phase blockade, mediated by the degradation of the AR-specific inhibitor of hormones. The degradation of AR specifically inhibits the proliferation of hormone-dependent prostate cancer cells (Rodriguez-Gonzalez et al., 2008). Cephalomannan, a small molecule compound, inhibits the activity of the UBE2S promoter (Zhang R. Y. et al., 2021). Xiao et al. demonstrated that UBE2S expression progressively decreases in a dose-dependent manner in cephalomannan-treated BCa cells. Mechanistically, cephalomannan inhibits BCa metastasis *in vitro* and *in vivo* in a dose-dependent manner by targeting UBE2S. Furthermore, cephalomannan reduces the expression of the mesenchymal marker N-calmodulin, suggesting that it effectively blocks EMT in BCa-like tissues (Xiao et al., 2023).

## Conclusion

Over the past few decades, significant efforts have been dedicated to elucidating the molecular mechanisms underlying urologic diseases. In this review, we present a comprehensive summary of the current research advancements regarding the roles of ubiquitination and deubiquitination in urinary diseases and their therapeutic strategies. While E3 ubiquitin ligases and DUBs have been shown to regulate processes associated with urinary diseases, several important issues warrant discussion. In several studies, researchers have identified a role for ubiquitination in the pathogenesis of urinary tract diseases. However, these studies do not specify a clear E3 ubiquitin ligase or deubiquitinase. For instance, silencing FAM46B enhances β-catenin protein expression by inhibiting its ubiquitination, which in turn promotes cell proliferation and cell cycle progression in PC (Liang et al., 2018). Additionally, n6-methyladenosine enhances the translation of ENO1 by inhibiting PCNA ubiquitination, thereby facilitating the progression of bladder cancer (Shen et al., 2024). Furthermore, SIRT3 mediates the ubiquitination and degradation of mitofusin 2, which inhibits ischemia-reperfusion-induced acute kidney injury (Shen et al., 2021). Although these studies suggest a significant role for ubiquitination in disease progression, they do not identify specific ubiquitinating enzymes. Similar to ubiquitination, other PTMs, including acetylation, methylation, phosphorylation, and SUMOylation, play significant roles in the progression of urologic diseases. Complex interactions and crosstalk among these various PTMs have been observed. For instance, research indicates that the ubiquitination and SUMOylation modifications of a target protein can be differentially regulated based on the specific stimulus or environmental context. This regulation can occur in a synchronized manner, where SUMOylation facilitates or dictates ubiquitin-mediated degradation; in a competitive manner, where SUMOylation inhibits ubiquitination; or in an independent manner, where SUMOylation has minimal impact on ubiquitination (Chen and Lu, 2015). Additionally, studies have demonstrated that NDR1/FBXO11 enhances phosphorylation-mediated ubiquitination of β-catenin, thereby inhibiting metastasis in prostate cancer (Xuan et al., 2024). Therefore, it is imperative to further investigate the mechanisms through which ubiquitination and deubiquitination contribute to the development of urological diseases. The role of UPS in urologic diseases has led to the identification of potential therapeutic targets, which have prompted further investigation into corresponding inhibitors. Notable examples include Carbidopa, yuanhuacine, Protac-A, and cefaglumine. Research is also focusing on targeted inhibitors of E1 enzymes, E2 enzymes, E3 ligases, deubiquitinases, and additional targets such as MDM2 inhibitors, IAP inhibitors, and SKP2 inhibitors (Ye et al., 2021). Consequently, multi-target combination therapy appears to be a promising direction for future research. To the best of our knowledge, this represents the first comprehensive report on the subject, aiming to provide an objective and thorough summary while exploring potential connections and offering insights for the future clinical development of relevant targeted drugs.
